# Fast frequency modulation is encoded according to the listener expectations in the human subcortical auditory pathway

**DOI:** 10.1162/imag_a_00292

**Published:** 2024-09-19

**Authors:** Alejandro Tabas, Stefan Kiebel, Michael Marxen, Katharina von Kriegstein

**Affiliations:** Basque Center on Cognition, Brain, and Language, San Sebastian, Spain; Ikerbasque, Basque Foundation for Science, Bilbao, Spain; Max Planck Institute for Human Cognitive and Brain Sciences, Leipzig, Germany; Department of Psychology, Technische Universität Dresden, Dresden, Germany; Department of Psychiatry, Technische Universität Dresden, Dresden, Germany; Neuroimaging Center, Technische Universität Dresden, Dresden, Germany

**Keywords:** predictive coding, auditory, sensory pathway, frequency modulation, fMRI, auditory midbrain, auditory thalamus, IC, MGB

## Abstract

Expectations aid and bias our perception. For instance, expected words are easier to recognise than unexpected words, particularly in noisy environments, and incorrect expectations can make us misunderstand our conversational partner. Expectations are combined with the output from the sensory pathways to form representations of auditory objects in the cerebral cortex. Previous literature has shown that expectations propagate further down to subcortical stations during the encoding of static pure tones. However, it is unclear whether expectations also drive the subcortical encoding of subtle dynamic elements of the acoustic signal that are not represented in the tonotopic axis. Here, we tested the hypothesis that subjective expectations drive the encoding of fast frequency modulation (FM) in the human subcortical auditory pathway. We used fMRI to measure neural responses in the human auditory midbrain (inferior colliculus) and thalamus (medial geniculate body). Participants listened to sequences of FM-sweeps for which they held different expectations based on the task instructions. We found robust evidence that the responses in auditory midbrain and thalamus encode the difference between the acoustic input and the subjective expectations of the listener. The results indicate that FM-sweeps are already encoded at the level of the human auditory midbrain and that encoding is mainly driven by subjective expectations. We conclude that the subcortical auditory pathway is integrated in the cortical network of predictive processing and that expectations are used to optimise the encoding of fast dynamic elements of the acoustic signal.

## 1 Introduction

Expectations can have dramatic effects on sensory processing (de Lange et al., 2018). A prime example is speech perception, where word recognition is strongly affected by semantic context (Davis et al., 2011), word prevalence (Sereno et al., 2003), and prior knowledge (Sohoglu et al., 2012). Predictive coding is one of the leading frameworks explaining how expectations affect perceptual encoding (Friston, 2003,^2005^;Rao & Ballard, 1999). A key hypothesis of the framework is that sensory neurons at lower levels do not encode the features of the stimuli but prediction error: the difference between the sensory input and the predictions of an internal generative model of the sensory world.

The encoding of prediction error to fast dynamic stimuli has been robustly demonstrated in the auditory cortex (Blank & Davis, 2016;Blank et al., 2018;Hovsepyan et al., 2020;Signoret et al., 2020;Sohoglu & Davis, 2020;Stein et al., 2022;Vidal et al., 2019;Ylinen et al., 2016). However, anatomical and physiological properties make the subcortical auditory pathway very well suited to test hypotheses on fast dynamic sounds (Giraud et al., 2000;Osman et al., 2018;von Kriegstein et al., 2008): Neural populations in the auditory midbrain (inferior colliculus; IC) and thalamus (medial geniculate body; MGB) are endowed with much shorter time constants and faster access to acoustic information than neural populations in the cerebral cortex (Steadman & Sumner, 2018). Moreover, the nuclei are the target of massive cortico-thalamic and cortico-collicular efferent systems (Lee & Sherman, 2011;Schofield, 2011;Winer, 1984,2005a) that are propitious to transmit complex predictions.

Stimulus-specific adaptation (SSA) has been used as a first attempt to test for predictive coding in the subcortical pathways. SSA is a phenomenon where individual neurons adapt to repetitions of a pure tone but show recovered responses to a frequency deviant (Ulanovsky et al., 2003). SSA is present in single neurons of the rodent’s IC (Ayala et al., 2015;Gao et al., 2014;Parras et al., 2017;Pérez-González et al., 2012;Robinson et al., 2016;Zhao et al., 2011) and MGB (Anderson et al., 2009;Antunes et al., 2010;Bauerle et al., 2011;Parras et al., 2017), and in neural populations of the human IC and MGB (Cacciaglia et al., 2015;Cornella et al., 2015;Escera & Malmierca, 2014;Grimm et al., 2011;Tabas et al., 2020). SSA can, however, be explained by both neural habituation and predictive coding (seeTabas & von Kriegstein, 2021afor a review). In the case of pure tones, we have recently used a novel SSA paradigm which revealed that SSA in human IC and MGB is driven largely by an internal model of the sensory world informed by the subjective expectations of the listeners, as hypothesised by predictive coding but not by neural habituation (Tabas et al., 2020).

In contrast to pure tones, natural sounds comprise highly dynamic elements that cannot be fully characterised by mixtures of pure tones. An ubiquitous example of these dynamic elements are fast frequency-modulated (FM)-sweeps (Liberman & Studdert-Kennedy, 1978;Liberman et al., 1956). While pure tones are encoded according to their frequency along the tonotopic axis already at the basilar membrane (Hu, 2003), FM-sweeps are encoded in FM-direction and FM-rate selective neurons (Kuo & Wu, 2012). In humans, the lowest level in the auditory hierarchy with evidence for fast FM-direction (Hsieh et al., 2012;Joanisse & DeSouza, 2014) and rate (Okamoto & Kakigi, 2015) selectivity is in auditory cortex; however, FM-sensitive neurons have been reported in the rodent IC and MGB (Issa et al., 2016;Kuo & Wu, 2012;Lui & Mendelson, 2003;Ye et al., 2010;Zhang et al., 2003). Here, we focus on the subcortical auditory pathway, where the encoding of FM has not been shown yet in humans, and where the encoding of FM as prediction error would be the most surprising.

We addressed two key questions. First, whether FM-rate and FM-direction are already encoded in neural populations of the human IC and MGB. Second, whether fast FM-sweeps are encoded in IC and MGB according to the principles of predictive coding; that is, as prediction error with respect to a generative model of the sensory world that incorporates the subjective expectations of the listener. We measured BOLD responses in the IC and MGB while participants listened to sequences of FM-sweeps. We used abstract rules to manipulate the subjective expectations of the participants on the incoming FM-sweeps independently of local stimulus statistics. We reasoned that, if FM-sweeps were encoded according to their objective properties, an FM-sweep embedded in a specific statistical context should elicit the same activation no matter the expectations that participants have on its occurrence. Reversely, if FM-sweeps were encoded according to the principles of predictive coding, BOLD responses should directly depend on how well the sensory input fits the expectations of the listeners.

## 2 Methods

This study was approved by the Ethics committee of the Technische Universtät Dresden, Germany (ethics approval number EK 315062019). All listeners provided written informed consent and received monetary compensation for their participation.

### 2.1 Participants

Eighteen German native speakers (12 female), aged 19 to 31 years (mean 24.6), participated in the study. None of them reported a history of psychiatric or neurological disorders, hearing difficulties, or current use of psychoactive medications. Normal hearing abilities were confirmed with pure tone audiometry (250 Hz to 8000 Hz); all participants had hearing threshold equal to or below 15 dB SPL in the frequency range of the stimuli used in the experiment (1000 Hz–3000 Hz). Participants were also screened for dyslexia (German SLRT-II test (Moll & Landerl, 2014), RST-ARR (Ibrahimović & Bulheller, 2013), and rapid automatised naming (RAN) test of letters, numbers, objects, and colours (Denckla & Rudel, 1974)) and autism (Autism Spectrum Quotient; AQ (Baron-Cohen et al., 2001)). All scores were within the neurotypical range (SLRT:$\text{min}\left(\text{max}\left(PR_{\text{words}},PR_{\text{pseudowords}}\right)\right)=21$, higher than the cut-off value of 16, following the same guidelines asGutschmidt et al. (2021); RST-ARR: allPR≥31, higher than the cut-off value of 16; RAN: maximum of 3 errors andRT<36seconds in each of the four categories; AQ: all participantsAQ≤31, under or equal to the cut-off value of 32).

Since we had no estimations of the possible sizes of the effects, we maximised our statistical power by recruiting as many participant as we could fit in the MRI measurement time allocated to the study. This number was fixed to 20 before we started data collection, but 2 participants dropped out of the study during data collection. We maximised the amount of data collected for participant to reduce random error to a minimum and maximise the likelihood of measuring effects at the single-subject level.

### 2.2 Stimuli

**Fig. 1.**
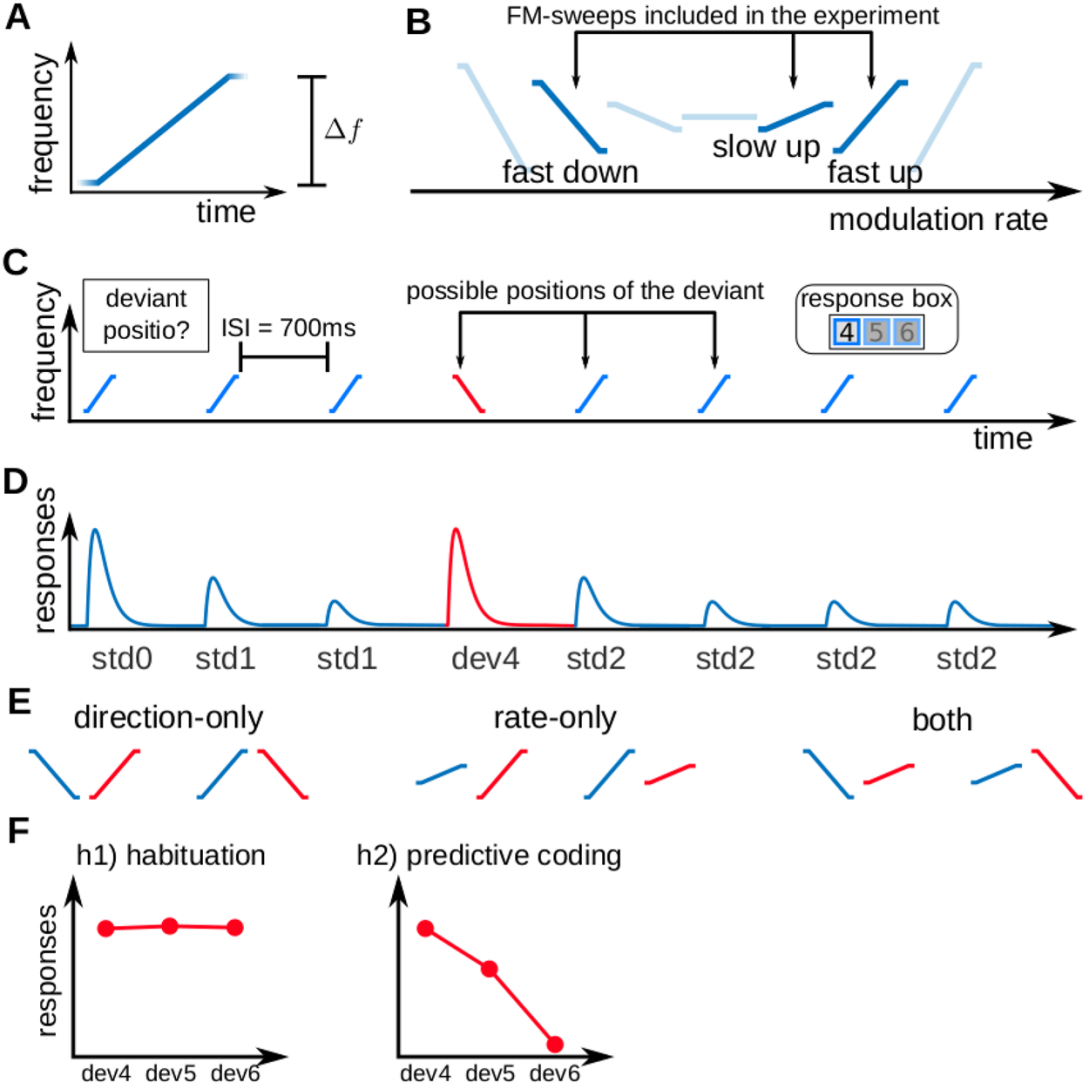
Experimental design and hypotheses. (A) Example of an FM-sweep with positive FM-rate. (B) The three FM-sweeps used in the experiment (in dark blue) in comparison to an hypothetical family of seven sweeps with increasing modulation rate. All sweeps had the same duration of 50 ms. They were characterised by differences in the frequency spanΔf. (C) Example trial. Each trial consisted of a sequence of seven repetitions of one FM-sweep (standards; blue) and one other FM-sweep (deviant; red). In each trial, a single deviant was located in positions 4, 5, or 6 of the sequence. Participants reported, in each trial, the position of the deviant right after they identified it. Each participant completed up to 540 trials in total, 60 per deviant position and FM-sweep combination$\Delta =\left|\Delta f_{\text{deviant}}-\Delta f_{\text{standard}}\right|$. Sweeps within a sequence were separated by 700 ms inter-stimulus-intervals (ISIs). (D) Schematic view of the expected underlying responses in the auditory pathway for the sequence shown in (C), together with the definition of the experimental variables (std0: first standard;std1: repeated standards preceding the deviant;std2: standards following the deviant;devx: deviant in positionx). (E) Schematic view of the six standard (blue) and deviant (red) combinations. Combinations are characterised by whether deviant and standard differ in: modulation direction only, modulation rate only, or both. (F) Expected responses in the auditory pathway nuclei corresponding to the hypotheses: h1) responses reflect adaptation by habituation only; h2) responses reflect prediction error with respect to the participant’s expectations.

The stimuli were three fast FM-sweeps: One sweep with a fast negative FM-rate (frequency spanΔf=−200Hz), one with a fast positive FM-rate (frequency spanΔf=200Hz), and one with a slow positive FM-rate (frequency spanΔf=100Hz;Fig. 1A, B). We used 50 ms long sweeps in the frequency range off∼1100Hz so that they had the typical properties of formant transitions in speech (Liberman & Studdert-Kennedy, 1978). The sweep average frequencies were adjusted so that all FM-sweeps were perceived as having the same pitch (Nabelek et al., 1970;Tabas & von Kriegstein, 2021b). We used a previous computational model to confirm that the selected FM-sweeps would elicit the same average activity along the tonotopic axis (Tabas & von Kriegstein, 2021b) and thus the same representation (averaged across the 50 ms duration of the stimuli) in the receptive fields encoding pure tones; that is, the receptive fields that were putatively used to differentiate between the stimuli inTabas et al. (2020). Thus, if the model predictions are correct, participants would needed to engage FM-direction and FM-rate selective circuits to differentiate between any two sweeps in the present paradigm.

All sounds were 50 ms long (including 5 ms in/out ramps) sinusoidal FM sweeps. The frequency sweeps lasted for 40 ms and were preceded and followed by 5 ms long segments of constant frequency that overlapped with the in/out ramps (Fig. 1A). The constant frequency and sweep segments were merged in the frequency space to avoid discontinuities in the stimulus waveforms. The constant frequency segments were used to guarantee that the entire sweep segments, which were controlled to elicit the same pitch percept, were played at the same loudness and audible in the loud noise generated by the MRI scanner. Participants could not have used these segments to characterise the sounds because they were only audible (having a loudness that was comparable or larger than the scanning noise) for about 2 ms, whereas the auditory system needs to integrate along four repetition cycles of the stimuli to characterise pitch (Pressnitzer et al., 2001); that is, for about 6 ms for a∼1.5kHz tone. Moreover, the constant frequency segments would be strongly affected by forward and backward masking (Elliott, 1971) from the louder and longer sweep segment.

We used a total of three sweeps during the experiment: a*fast up*sweep with starting frequency$f_{0}=1000$Hz and ending frequency$f_{1}=1200$Hz (Δf=200Hz); a*slow up*sweep with$f_{0}=1070$Hz and$f_{1}=1170$Hz (Δf=100Hz), and a fast down sweep with$f_{0}=1280$Hz and$f_{1}=1080$Hz (Δf=−200Hz). The sweep average frequencies were adjusted so that all FM-sweeps elicited the same average activity along the tonotopic axis and were perceived as having the same pitch (Nabelek et al., 1970;Tabas & von Kriegstein, 2021b); this design guaranteed that FM-direction and FM-rate selective neurons were necessary to differentiate between any two sweeps in the paradigm.

### 2.3 Experimental paradigm

We arranged the stimuli in sequences of 8 FM-sweeps with 7 repetitions of the same sweep (standard) and one deviating sweep (deviant) (Fig. 1C). Participants were instructed to report, with a button press, the position of the deviant within the sequence as fast and accurately as possible after identifying the deviant. Each sequence was characterised by the position of the deviant and the combination of sweeps. There were three combinations (Fig. 1E): one where the sweeps differed only on modulation direction, one where the sweeps differed only on modulation rate, and one where the sweeps differed in both.

In each trial of the fMRI experiment, participants listened to one tone sequence and reported,*as fast and accurately as possible*using a button box with three buttons, the position of the deviant (4, 5 or 6). The inter-trial-interval (ITI) was jittered to maximise the efficiency of the response estimation of the deviants (Friston et al., 1999). To do this, we first sampled the time lapsed between deviants (inter-deviant-interval; IDI) from a truncated normal distribution with an average of 5 seconds and a standard deviation of 1 second, truncated between 3 and 11 seconds. We used the deviant position of the current and next trial to compute the corresponding ITI given the sampled IDI, and further constrained the ITI to be of a minimum of 1.5 seconds to ensure that participants were able to tell consecutive trials apart from each other. Participants were allowed to report the deviant position up to 2000 ms after the offset of the last tone; if participants had not responded after the minimum ITI of 1500 ms, this minimum was automatically extended to accommodate the additional waiting period. This construction resulted in the following summary statistics for the IDIs: mean of 7.2 seconds, minimum of 5.2 seconds, maximum of 13.0 seconds; and the following summary statistics for the ITIs: minimum of 1.5 seconds, mean of 4.8 seconds. These summary statistics ignored the periods of silence corresponding to the null trials.

We implicitly modulated the participant’s subjective expectations on the incoming stimuli using two abstract rules that were disclosed to the participants: 1) all sequences have a deviant, and 2) the deviant is always located in position 4, 5, or 6. The three deviant positions were used the same number of times along the experiment, so that the three deviant positions were equally likely at the beginning of the sequence. Therefore, the likelihood of finding a deviant in position 4 after hearing 3 standards is1/3. However, if the deviant is not located in position 4, it must be located in either position 5 or 6, so participant expect a deviant in position 5 after hearing 4 standards with a probability1/2. The probability of finding a deviant in position 6 after hearing 5 standards is1.

### 2.4 Experimental design

Participants completed the task while we measured BOLD responses in participants’ IC and MGB with an fMRI-sequence. All but one participant completed 9 runs of the main experiment across three sessions; participant 18 completed only 8 runs for technical reasons.

Each run contained 6 blocks of 10 trials. The 10 trials in each block used one of the 6 possible sweep combinations, so that all the sequences within each block had the same standard and deviant. Thus, within a block only the position of the deviant was unknown, while the deviant’s FM-direction and FM-rate were known in nine of every 10 trials. The order of the blocks within the experiment was randomised. The position of the deviant was pseudorandomised across all trials in each run so that each deviant position happened 180 times per participant but an unknown amount of times per run. This constraint allowed us to keep the same a priori probability for all deviant positions in each block. In addition, there were 23 silent gaps of 5300 ms duration (i.e., null events of the same duration as the tone sequences) randomly located in each run (Friston et al., 1999), which did not necessarily fall at the end or beginning of a block. Each run lasted around 10 minutes, depending on the reaction times of the participant.

Due to an undetected bug in the presentation code, the standard/deviant combination of the trial was incorrectly recorded in some runs. The bug affected the first three runs of participants 1, 2, 4, and 5; and the first six runs of participant 3. This information was not relevant for the analyses that aggregated the data across sweep combinations, and affected only the analyses ofFigure 5, where we excluded the affected runs of participants 1, 2, 4, and 5, and participant 3 altogether.

### 2.5 Functional localiser

We also run a functional localiser that was designed to activate the participant’s IC and MGB. Each run of the functional localiser consisted of 20 blocks of 16 seconds and lasted for about 6.5 minutes. Ten of the blocks were silent; the remaining blocks consisted of presentations of 16 contiguous sounds of 1 second duration each. Sounds were taken from a collection of 85 natural sounds collected byMoerel et al. (2015). Participants were instructed to press a key when the same sound was repeated twice, which happened on 5% of the trials. The participants received this task to ensure that they attended the sounds: behavioural data from the functional localiser was not used in the analysis.

### 2.6 Experiment structure

Each session consisted of three runs of the main experiment, interspersed with two runs of the functional localiser. All runs were separated by breaks of a minimum of 1 minute to allow the participants rest. Fieldmaps and a whole-head EPI were acquired between the third and fourth run. In the first session, we also measured a structural image before the fieldmaps. The first run of the first session was preceded by a*practice run*of four randomly chosen trials to ensure the participants had understood the task. We acquired fMRI during the practice run in order to allow the participants to undertake the training with MRI-noise.

### 2.7 Data acquisition

MRI data were acquired using a Siemens Trio 3 T scanner (Siemens Healthineers, Erlangen, Germany) with a 32-channel head coil. Functional MRI data were acquired using echo planar imaging (EPI) sequences. We used partial coverage with 24 slices. The volume was oriented in parallel to the superior temporal gyrus such that the slices encompassed the IC, the MGB, and the superior temporal gyrus. In addition, we acquired one volume of an additional whole-head EPI with the same parameters (including the FoV) and 84 slices during resting to aid the coregistration process (seeSection 2.8).

The EPI sequence had the following acquisition parameters: TR = 1900 ms, TE = 42 ms, flip angle 66°, matrix size88×88, FoV 154 mm×154 mm, voxel size 1.75 mm isotropic, bandwidth per pixel1386Hz/px, and interleaved acquisition. During functional MRI data acquisition, cardiac signal was acquired using a scanner pulse oximeter (Siemens Healthineers, Erlangen, Germany).

Structural images were recorded using an MPRAGE (Brant-Zawadzki et al., 1992) T1 protocol with 1 mm isotropic resolution, TE = 1.95 ms, TR = 1000 ms, TI = 880 ms, flip angle 1 = 8°, and FoV = 256 mm×256 mm.

Stimuli were presented using MATLAB (The Mathworks Inc., Natick, MA, USA) with the Psychophysics Toolbox extensions (Brainard, 1997) and delivered through an Optoacoustics (Optoacoustics Ltd, Or Yehuda, Israel) amplifier and headphones equipped with active noise-cancellation. Loudness was adjusted independently for each participant to a comfortable level before starting the data acquisition.

### 2.8 Data preprocessing

The preprocessing pipeline was coded in Nipype 1.5.0 (Gorgolewski et al., 2011), and carried out using tools of the Statistical Parametric Mapping toolbox, version 12; Freesurfer, version 6 (Fischl et al., 2002); the FMRIB Software Library, version 5 (FSL) (Jenkinson et al., 2012)); and the Advanced Normalization Tools, version 2.3 (ANTS) (Avants et al., 2011). All data were coregistered to the Montreal Neurological Institute (MNI) MNI152 1 mm isotropic symmetric template.

First, we realigned the functional runs. We used SPM’s*FieldMap Toolbox*to calculate the geometric distortions caused in the EPI images due to field inhomogeneities. Next, we used SPM’s*Realign and Unwarp*to perform motion and distortion correction on the functional data. Motion artefacts, recorded using SPM’s ArtifactDetect, were later added to the design matrix (seeSection 2.9).

Next, we used Freesurfer’s recon-all routine to calculate the boundaries between grey and white matter (these are necessary to register the functional data to the structural images) and ANTs to compute the transformation between the structural images and the MNI152 symmetric template.

Last, we coregistered the functional data to the structural image with Freesurfer’s*BBregister*, using the boundaries between grey and white matter of the structural data and the whole-brain EPI as an intermediate step. Data were analysed in the participant space, and then coregistred to the MNI152 template. Note that, since the resolution of the MNI space (1 mm isotropic) was higher than the resolution of the functional data (1.75 mm isotropic), the transformation resulted in a spatial oversampling.

All the preprocessing parameters, including the smoothing kernel size, were fixed before we started fitting the general linear model (GLM) and remained unchanged during the subsequent steps of the data analysis.

Physiological (heart rate) data were processed by the PhysIO Toolbox (Kasper et al., 2017), that computes the Fourier expansion of each component along time and adds the coefficients as covariates of no interests in the model’s design matrix.

### 2.9 Estimation of the BOLD responses

First level analyses were coded in Nipype and carried out using SPM. Second-level analyses were carried out using custom code in MATLAB. The coregistered data were first smoothed using a 2 mm FWHM Gaussian kernel with SPM’s*Smooth*.

The first-level GLM’s design matrix for the main experiment included 6 regressors: first standard (std0), standards before the deviant (std1), standards after the deviant (std2), and deviants in positions 4, 5, and 6 (dev4, dev5, and dev6, respectively;Fig. 1). Conditions std1 and std2 were modelled using linear parametric modulation (O’Doherty et al., 2007), whose linear factors were coded according to the position of the sound within the sequence to account for effects of habituation (Tabas et al., 2020;Supplementary Fig. S1). The first-level GLM’s design matrix for the functional localiser included 2 conditions: sound and silence. On top of the main regressors, the design matrix also included the physiological PhysIO and artefact regressors of no-interest. Beta values werez-scored per run and participant before running group statistics to ensure they all had zero mean and unit variance (Devore, 2008).

This design allowed us to maximally disentangle responses to stimuli that were close to each other in time at the cost of introducing the reasonable assumption that the responses to the repeated standards (std1andstd2) varied approximately linearly across successive repetitions. The resulting design matrix, convoluted by the hemodynamic response function (Glover, 1999), presents moderate correlations between most pairs of regressors (Supplementary Fig. S2). Although these correlations reduce the statistical power to detect differences in responses to correlated regressors, with over 360 minutes of measured data for the main task per participant, our study is well equipped to compensate for the resulting decrease on statistical power. Moreover, correlation between regressors can under no circumstance result in an increase of type I errors (i.e., false positives) (Mumford et al., 2015); therefore, the measured correlations do not challenge the interpretability of positive results.

Analyses geared towards testing whether responses to different deviants differed were carried out by fitting the regressors across all trials to maximise statistical power. Analyses geared towards testing specific sensitivity to FM-direction or FM-rate were carried out by defining a total of 18 regressors, 6 for each of the three standard/deviant combinations (Fig. 1E).

### 2.10 Definition of the anatomical and SSA ROIs

We used a recent anatomical atlas of the subcortical auditory pathway (Sitek et al., 2019) to compute prior regions corresponding to the left IC, right IC, left MGB, and right MGB, respectively. The atlas comprises three different definitions of the ROIs calculated using 1) data from the big brain project, 2) postmortem data, and 3) fMRI in vivo-data. To compute the prior coarse region for each nuclei, we combined the three masks and inflated the resulting regions with a Gaussian kernel with FWHM = 1 mm isotropic. Next, we used SPM to compute the contrast*sound*>*silence*of the data from the functional localiser. We then masked this contrast with each of the prior coarse regions. Last, we iteratively thresholded the contrast to increasingly conservative higher values until the number of surviving voxels equal the volume of the region reported inSitek et al. (2019); namely, 146 voxels for each of the ICs, and 152 for each of the MGBs.

The final IC and MGB regions were computed by combining the prior coarse regions with the results from the contrastsound>silenceof the functional localiser. Within each region, we thresholded the contrast to increasingly higher values until the number of surviving voxels equalled the volume of the region reported inSitek et al. (2019); namely, 146 voxels for each of the ICs, and 152 for each of the MGBs.

To address our first research question, whether neural populations of human IC and MGB encode FM-rate and FM-direction, we tested whether these two nuclei show SSA to the FM-sweeps used in the experiment; namely, if neural responses in IC and MGB adapt to repeated FM-sweeps while preserving high responsiveness to FM-sweeps that deviate from the standards in FM-rate or FM-direction (Fig. 1D). Since all sweeps were designed to elicit the same average activation across the tonotopic axis and elicited the same pitch percept, neural populations showing SSA to these FM-sweeps necessarily comprise neurons that are sensitive to FM-rate and FM-direction.

We used the coefficients of the GLM or beta estimates from the first-level analysis to calculate the adaptation and deviant detection ROIs, defined as the sets of voxels within the IC and MGB ROIs that responded significantly to the contrastsstd0>0.5 std1+0.5 std2anddev4>0.5std1+0.5std2, respectively. Significance was defined asp<0.05, family-wise-error (FDR)-corrected for the number of voxels within each of the IC/MGB ROIs. SSA voxels are defined as voxels that show both, adaptation and deviant detection; thus, we calculated an upper bound of thep-value maps for the SSA contrast as the maximum of the uncorrectedp-values associated to the adaptation and deviant detection contrasts. The SSA ROIs were calculated by FDR-correcting and thresholding the resultingp-maps atα=0.05. All calculations were performed using custom-made scripts (seeData and Code Availability section).

### 2.11 Bayesian model comparison

To address our second research question, whether IC and MGB responses encode FM-sweeps as prediction error with respect to the listener expectations, we used Bayesian model comparison. According to predictive coding, both the responses to deviants and standards should scale with the predictability of the stimuli. Due to the limited temporal resolution of fMRI, we cannot use a classical analysis to robustly estimate the responses to the standards and the deviants simultaneously in each single voxel. However, by introducing reasonable assumptions on the response patterns expected by habituation (Malmierca et al., 2009) and prediction error (Friston, 2003), Bayesian techniques can evaluate whether a voxel is significantly likely to encode prediction error to both, deviants and standards.

We considered two models. The first model assumed that adaptation to repeated fast FM-sweeps was driven by habituation to the stimulus sequence properties, independently of participant’s expectations; namely, that neural populations habituate to repetitions of the standard, but show recovered responses to deviant irregardless of their position (habituation hypothesis;Fig. 1F,h1). The second model assumed that adaptation was driven by predictive coding; namely, that neural responses to the deviants encoded prediction error with respect to the expectations of the participants (predictive coding hypothesis;Fig. 1F,h2). Although we expect habituation to also contribute to the BOLD response in this last scenario, we conservatively decided not to include an additional habituation regressor in h2 to limit its explanatory power in voxels that are not driven by prediction error.

The Bayesian analysis of the data consisted as well of first- and second-level analyses. In the first level, we used SPM via nipype to compute the log-evidence in each voxel of each participant for each of the four models (seeFig. 2). The models were described using regressors with parametric modulation whose coefficients corresponded to a simplified view of the expected responses according to each model (Table 1). The expected responses of each model were the same in all trials that had the same standard-deviant combination and deviant position. Given the model amplitude(s)$a_{n}$and the timecourse of a voxely, SPM calculates the log-evidence of the linear model$y=\sum \beta_{n}a_{n}+\xi$, where$\beta_{n}$are the linear coefficients of each regressor andξare noise terms.

**Table 1.** Amplitudes of the models used for Bayesian Model Comparison.

	1	2	3	4	5	6	7	8	
h1	deviant in 4	$a_{0}$	$a_{1}$	$a_{1}\;/\;2$	$a_{2}$	$a_{3}$	$a_{3}\;/\;2$	$a_{3}\;/\;3$	$a_{3}\;/\;4$
	deviant in 5	$a_{0}$	$a_{1}$	$a_{1}\;/\;2$	$a_{1}\;/\;3$	$a_{2}$	$a_{3}$	$a_{3}\;/\;2$	$a_{3}\;/\;3$
	deviant in 6	$a_{0}$	$a_{1}$	$a_{1}\;/\;2$	$a_{1}\;/\;3$	$a_{1}\;/\;4$	$a_{2}$	$a_{3}$	$a_{3}\;/\;2$
h2	deviant in 4	$a_{0}$	0	0	$2a_{1}\;/\;3$	0	0	0	0
	deviant in 5	$a_{0}$	0	0	$a_{1}\;/\;3$	$a_{1}/2$	0	0	0
	deviant in 6	$a_{0}$	0	0	$a_{1}\;/\;3$	$a_{1}/2$	0	0	0

H1 assumes an asymptotic decay ($a_{0}\propto 1/\;n$wherenis the position of the stimulus in the sequence) in the responses for all standards, a full response to deviants, and a recovery from the last standard before the deviant and the first standard after the deviant that is sufficient to make the responses to both standards comparable. The model was built as a simplification of the average dynamics reported in the animal literature on SSA (e.g.,Malmierca et al., 2009). The free parameters encode the relative decay from the first to the second standard ($a_{0}\;/\;a_{1}$), the recovery between the last standard before the deviant and the first standard after the deviant (modulated by$a_{3}$), and the recovery of the responses to the deviant ($a_{2}$). H2 assumes that the responses scale with predictability ($a_{0}=1-p$, wherepis the likelihood of finding the heard stimuli in each position). The model was built following the precision-weighted formulation of predictive coding (Friston, 2003), which assumes that predictability is a multiplicative factor in the generation of prediction error. We used an additional free parameter to encode the amount of prediction error elicited by the first standard ($a_{0}$, which, unlike the rest of the stimuli in the trial, is additionally affected by uncertainty in the time onset). These models include a larger number of free parameters than the ones we used in our previous study (Tabas et al., 2020). The additional parameters allowed us to capture a variety of habituation and prediction error dynamics within the same model, rendering the definitions more general. However, using the more restricted models fromTabas et al. (2020)yields similar results (Supplementary Fig. S2).

**Fig. 2.**
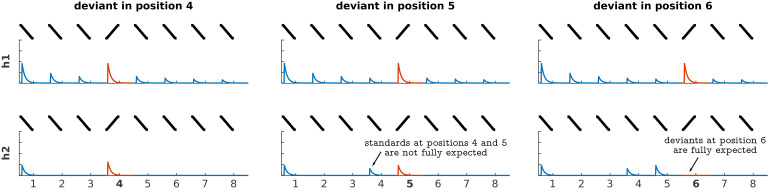
Design of the Bayesian models. The table shows the parametrised expected response to each tone in the sequence (rows) for the two different models (h1/h2) and the three deviant positions. Each model was defined according to the relative amplitudes it predicted for the different sounds in the sequences. H1 assumed asymptotic habituation to consecutive standards and recovered responses to deviants. H2 assumed that responses to the stimuli depended on how predictable they were. Note that the models have free linear parameters: the displayed amplitudes are one of the many possible solutions of the linear fit. SeeTable 1for an exact definition of each model.

Log-evidence maps for each participant were combined following the random-effects-equivalent procedure described inRosa et al. (2010)andStephan et al. (2009)to compute the posterior probability maps associated to each model at the group level. We combined the maps using custom scripts (seeData and Code Availability section). Histograms shown inFigures 7and8are kernel-density estimates computed with the distribution of the posterior probabilities across voxels for each of the SSA ROIs.

### 2.12 Statistical analysis

All pairwise comparisons reported in the study were evaluated for significance using two-tailed Ranksum tests. Unless stated otherwise,p-values for all analyses that comprised multiple testing were corrected using the Holm-Bonferroni method. A result was deemed statistically significant when the correctedp<0.05.

## 3 Results

### 3.1 Behavioural responses

**Fig. 3.**
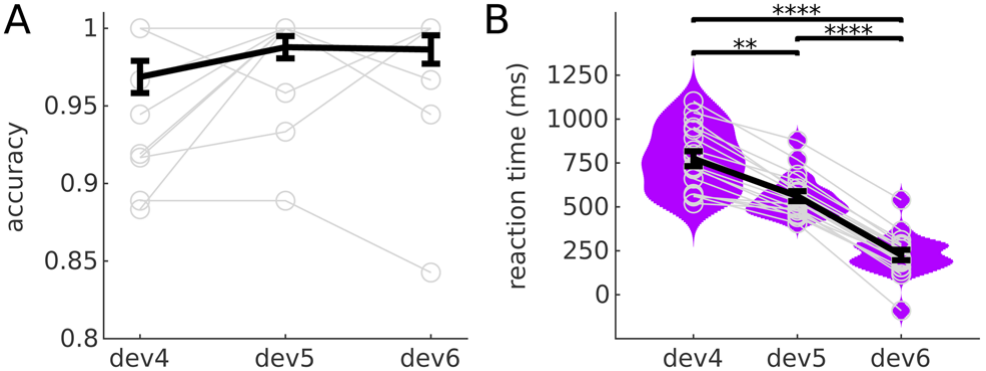
Performance and reaction times. Mean accuracy (A) and reaction times (B) across deviant positions. Grey circles represent the average value per participant and deviant position. Violin plots are the kernel density estimations of the reaction times for each deviant position. **p<0.005, ****p<0.00005; allp-values corrected for 3 comparisons.

Behavioural results showed an average accuracy over 0.96 to all deviant positions (Fig. 3A). Accuracy was slightly higher for the two more expected deviant positions, but differences between conditions were not significant (p>0.1, uncorrected). Reaction times (Fig. 3B) showed a behavioural benefit of expectations: Participants reacted faster to more expected deviants (averageRT=770ms,558ms and246ms for deviants at positions 4, 5, and 6, respectively; all differences were significant withp<0.0001, corrected for 3 comparisons).

### 3.2 Human IC and MGB show stimulus specific adaptation (SSA) to FM-sweeps

We first studied whether the IC and MGB show SSA to fast FM-sweeps to test if the two nuclei are sensitive to FM-rate and FM-direction in humans. To compute SSA, we determined which voxels within the ICs and MGBs adapted to the standard (i.e., adaptation) and recovered responsiveness to deviants (i.e., deviant detection). SSA regions were then defined as the intersection between adaptation and deviant detection regions. ICs and MGBs were identified based on structural MRI data and an independent functional localiser (seeSection 2; IC and MGB ROIs; coloured patches inFig. 4). Within these ROIs, we used non-parametric ranksum tests (N=18; one sample per participant) to find which voxels showed significant adaptation to repeated standards (contraststd0>0.5std1+0.5std2). The associatedp-maps were thresholded so that the false-discovery-rateFDR<0.05. Surviving voxels constituted the*adaptation*ROIs (blue and purple patches inFig. 4). The same procedure was used to delimit the*deviant detection*ROIs (red and purple patches inFig. 4): the set of voxels within each anatomical ROI that responded significantly stronger to deviants than to repeated standards (contrastdev4>0.5std1+0.5std2; note that we compare the responses to the repeated standards withdev4as this is the deviant position for which participants have the lowest expectation). The four anatomical ROIs showed significant adaptation (peakp≤0.0001) and deviant detection (peakp<0.0001; cluster size, exact peakp-values, and MNI coordinates are shown inTable 2; allp-values corrected for four comparisons).

**Table 2.** Statistics and MNI coordinates of the adaptation and deviant detection contrasts in the four regions of interest.

Contrast	ROI	Cluster size	MNI coordinates (mm)	Peak-level p -value
adaptation	left IC	130 voxels	[−4,−35,−9]	$p=1\times 10^{-4}$
	right IC	124 voxels	[4,−35,−9]	$p=8\times 10^{-5}$
	left MGB	152 voxels	[−14,−25,−7]	$p=8\times 10^{-5}$
	right MGB	146 voxels	[14,−26,−6]	$p=1\times 10^{-4}$
deviant detection	left IC	92 voxels	[−6,−33,−10]	$p=9\times 10^{-5}$
	right IC	91 voxels	[6,−33,−8]	$p=7\times 10^{-5}$
	left MGB	136 voxels	[−14,−24,−7]	$p=5\times 10^{-5}$
	right MGB	140 voxels	[11,−27,−5]	$p=2\times 10^{-5}$
SSA	left IC	91 voxels	[−4,−35,−9]	$p=3\times 10^{-4}$
	right IC	91 voxels	[6,−33,−9]	$p=2\times 10^{-4}$
	left MGB	136 voxels	[−14,−25,−7]	$p=2\times 10^{-4}$
	right MGB	140 voxels	[12,−26,−5]	$p=1\times 10^{-4}$

Allp-values FDR-corrected for the number of voxels in each anatomical ROI and further corrected for 4 comparisons within each contrast.

**Fig. 4.**
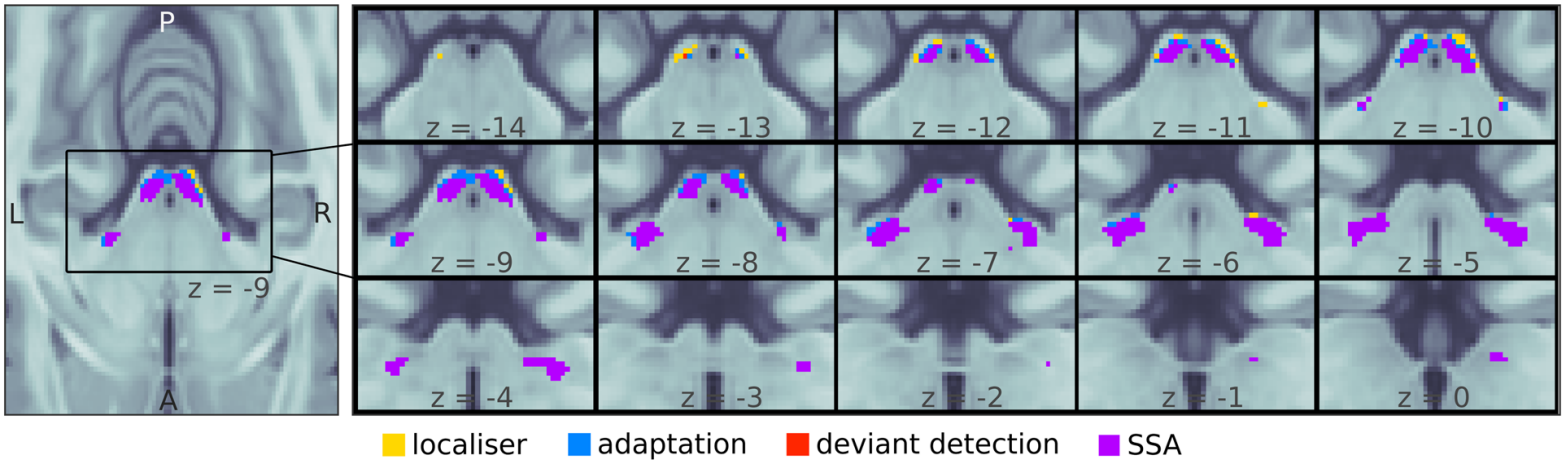
Mesoscopic stimulus specific adaptation (SSA) in bilateral IC and MGB. Regions within the MGB and IC ROIs adapted to the repeated standards (adaptation; blue shows adaptation only, purple shows SSA, which includes adaptation) and recovered responses to deviants (deviant detection; red shows deviant detection only, purple shows SSA, which includes deviant detection). Stimulus-specific adaptation (i.e., recovered responses to a deviant in voxels showing adaptation; SSA) occurred in bilateral MGB and IC (purple). Maps were computed by thresholding the contrastp-maps atFDR<0.05. Yellow patches show voxels included in the anatomical masks computed with a functional localiser that showed neither adaptation nor deviant detection.

SSA regions were computed combining the unthresholded*adaptation*and*deviant detection*p-maps. The uncorrectedp-value associated to SSA for a given voxel was$p_{\text{SSA}}=\text{max}\left(p_{\text{adaptation}},p_{\text{deviant detection}}\right)$. SSAp-maps where thresholded toFDR<0.05to compute the*SSA*ROIs (Fig. 4, purple). The four anatomical ROIs had extensive SSA regions (cluster sizes larger than 90$mm^{3}$; peakp≤0.0003; exact peakp-values and MNI coordinates are shown inTable 2; allp-values corrected for four comparisons).

Significant SSA was also found in at least one of the nuclei of 15 of the 18 participants (p≤0.048for each of the 15 participants, corrected for the 596 voxels included in a global subcortical auditory ROI that comprised bilateral IC and MGB), but not all participants showed significant SSA in all ROIs (IC-L: 8 participants,p≤0.049; IC-R, MGB-L, MGB-R: 6 participants each, withp≤0.048; allp-values corrected for the number of voxels in the ROI and further corrected for four ROIs).

These results confirmed that there are extensive regions of bilateral IC and MGB that selectively habituate, and therefore are sensitive, to FM direction and rate.

### 3.3 Human IC and MGB are sensitive to FM-direction and FM-rate

In the next step, we specifically tested whether the IC and MGB are similarly sensitive to FM-rate and FM-direction. To do that, we analysed the regressor fits corresponding to: 1) trials where the standard and deviant differed only in modulation direction but not in absolute modulation rate; and 2) trials where the standard and deviant differed only in modulation rate but not in direction. If IC and MGB encode direction and rate, we would expect similar results in both partitions of the data. Conversely, if human IC and MGB are only sensitive to one of the two properties, we would expect null effects in the partition of the data where the standard and deviants differ in the other property.

**Fig. 5.**
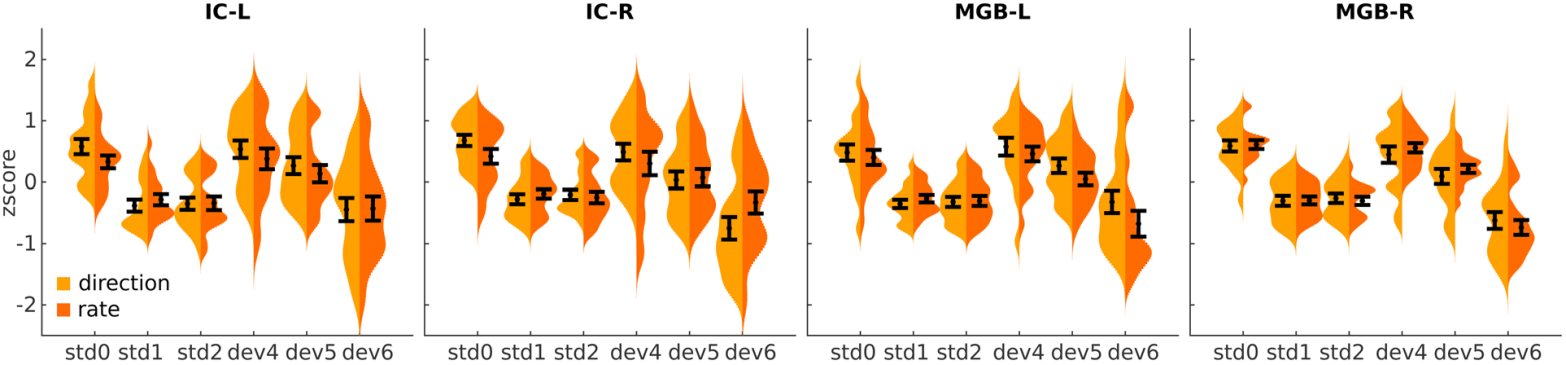
Summary BOLD responses for partitions of the data where deviant and standard differed only in direction or rate. Averagez-score in each of the four SSA ROIs to the different experimental conditions in trials where the standard and deviant differed only in direction (orange) or rate (yellow). Violin plots are kernel density estimations of the distribution ofz-scores, averaged over voxels and runs of each ROI. Each distribution holds 17 samples, one per participant (one participant was excluded from this analysis because there were not enough trials available, seeSection 2for details). Black error bars show the mean and standard error of the distributions.

Results were similar in both partitions of the data (Fig. 5), demonstrating that the human IC and MGB encode both FM-direction and FM-rate.

We further corroborated that the levels of SSA were comparable for both types of FM changes at the single-subject level. In order to characterise FM-sensitivity with a number for each subject and FM-sweep combination, we used the SSA index (Ulanovsky et al., 2003)SI(Eq. (1); note thatSI>0is equivalent to the deviant detection contrast used inFig. 4).

$$SI=\frac{dev4-\frac{1}{2}\left(std1+std2\right)}{dev4+\frac{1}{2}\left(std1+std2\right)}$$ (1)

We measured the difference inSIto FM-direction ($SI_{dir}$) and FM-rate ($SI_{rate}$) in the voxels of the subject-specific SSA regions calculated in the previous section for each of the 15 subjects for which we obtained significant SSA. If FM-direction and FM-rate are both encoded in IC and MGB, we would expect no difference between these two partitions of the data. We measured the difference using Cohen’s$d=\left(\langle SI_{dir}\rangle -\langle SI_{rate}\rangle \right)/\sigma$), where〈SI〉is the average ofSIandσis the pooled standard deviation. The difference ranged betweend≥−0.33andd≤0.475across participants. The expected value of the difference (E[d]=0.02±0.05) overlapped with zero, indicating once again that both FM-direction and FM-rate are already encoded in the subcortical auditory pathway.

### 3.4 Expectations drive the encoding of FM-sweeps in IC and MGB

**Fig. 6.**
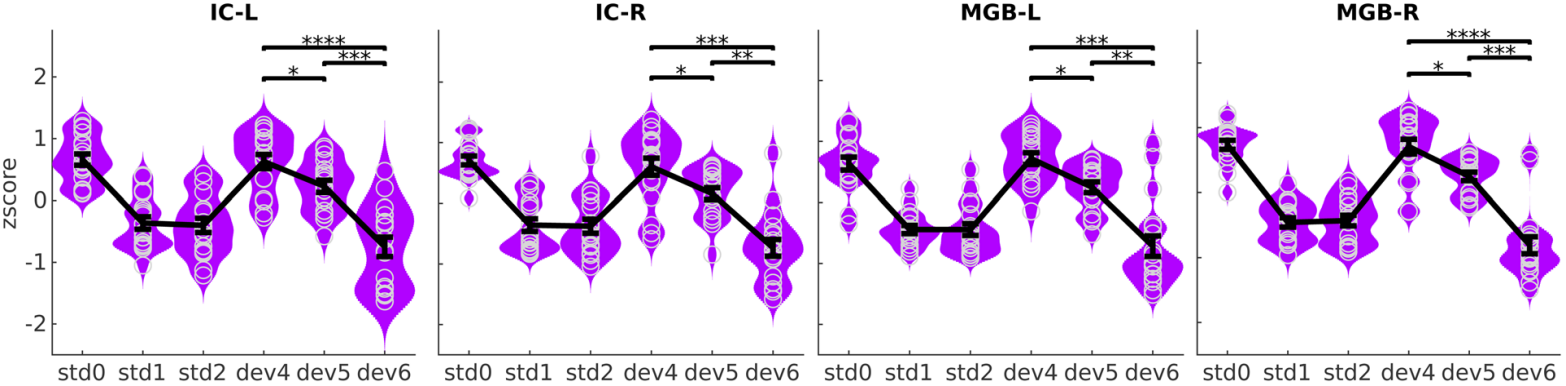
Summary BOLD responses. Averagez-score in each of the four SSA ROIs to the different regressors. Violin plots are kernel density estimations of the distribution ofz-scores, averaged over voxels and runs of each ROI. Each distribution holds 18 samples, one per participant. Black error bars show the mean and standard error of the distributions. Significance bars were computed by pooling across standard-deviant combinations. Single-subject distributions are shown inSupplementary Figure S4.Std0, first standard;std1: standards preceding the deviant;std2: standards following the deviant;dev4,dev5, anddev6: deviants at positions 4, 5, and 6, respectively (Fig. 1D). *p<0.05, **p<0.005, ***p<0.0005, ****p<0.00005; allp-values corrected for 12 comparisons.

To address our second question, we evaluated whether the average pooled BOLD responses to deviants in the three different positions were affected by participant’s subjective expectations within the SSA regions. In congruence with the predictive coding hypothesis (Fig. 1F,h2), the response profile showed reduced responses for more expected deviants (Fig. 6). This pattern was systematically reproduced in all subjects (Supplementary Fig. S4).

Formal statistical testing confirmed that responses to different deviant positions were different in all ROIs for all contrasts among deviant positions:dev4≠dev5(|d|≥0.99andp<0.006),dev4≠dev6(|d|≥2.39andp<0.00005), anddev5≠dev6(|d|≥1.74andp<0.0003; allp-values corrected for3×412 comparisons). Exactp-values and effect sizes are listed inTable 3. All statistical tests included one sample per participant, ROI, and deviant position.

**Table 3.** Statistics of the average BOLD response differences between deviant positions.

**IC-L**				
	dev5		dev6	
dev4	d=0.89	p=0.017	d=2.35	$p=4\times 10^{-5}$
dev5			d=1.75	$p=4\times 10^{-4}$
**IC-R**				
	dev5		dev6	
dev4	d=0.86	p=0.022	d=2.24	$p=10^{-4}$
dev5			d=1.74	$p=5\times 10^{-4}$
**MGB-L**				
	dev5		dev6	
dev4	d=1.20	p=0.0075	d=2.46	$p=10^{-4}$
dev5			d=1.68	p=0.0015
**MGB-R**				
	dev5		dev6	
dev4	d=1.17	p=0.0073	d=2.91	$p=2\times 10^{-5}$
dev5			d=2.40	$p=3\times 10^{-4}$

Effect size is expressed as Cohen’sd. Statistical significance was evaluated with two-tailed Ranksum tests between the distributions of the mean response in each ROI across participants (N=18), pooling across standard-deviant combinations. Allp-values in the table are corrected for3×4=12comparisons.

To corroborate that differences were present at the single-subject level, we run a correlation analysis for each of the 15 participants for which we obtained significant SSA. In each participant, we computed the Pearson’s correlation between the BOLD responses elicited by each deviant position with its likelihood of occurrence (namely,1/3for deviant 4,1/2for deviant 5, and1for deviant 6). If BOLD responses reflect prediction error, we would expect a negative correlation between the likelihood and the responses. We found significantly negative correlations in all 15 participants (ρ∈[−0.87,−0.42], allp<0.03; all Pearson tests had9×3=27samples, 3 per run).

### 3.5 FM-sweeps are encoded as prediction error in the majority of IC and MGB voxels

We used Bayesian model comparison to formally evaluate whether the responses in each voxel of the IC and MGB ROIs were best explained as prediction error. This approach provides for a quantitative assessment of the likelihood that each of the two hypotheses (Fig. 1F) can explain the responses in each voxel. This analysis is sensitive to possible region-specific effects that could have been averaged out when aggregating thez-scores across voxels in each ROI.

Following the methodology described inRosa et al. (2010)andStephan et al. (2009), we first calculated the log-likelihood of each model in each voxel of the two ICs and MGBs in each participant. Each model yields different predictions on the relative amplitudes to different positions in the sequences (Fig. 1F). We tested h1 and h2 to adjudicate between the habituation and predictive coding explanations of the responses. H1 assumed an asymptotic decay of the standards and recovered responses to the deviants; h2 assumed that the responses to both deviants and standards would depend on the participant’s expectations (Fig. 2; for exact values, seeSection 2). Participant-specific log-likelihoods were used to compute the Bayes’ factorK(i.e., the ratio of the posterior likelihoods) between h1 and h2.

**Fig. 7.**
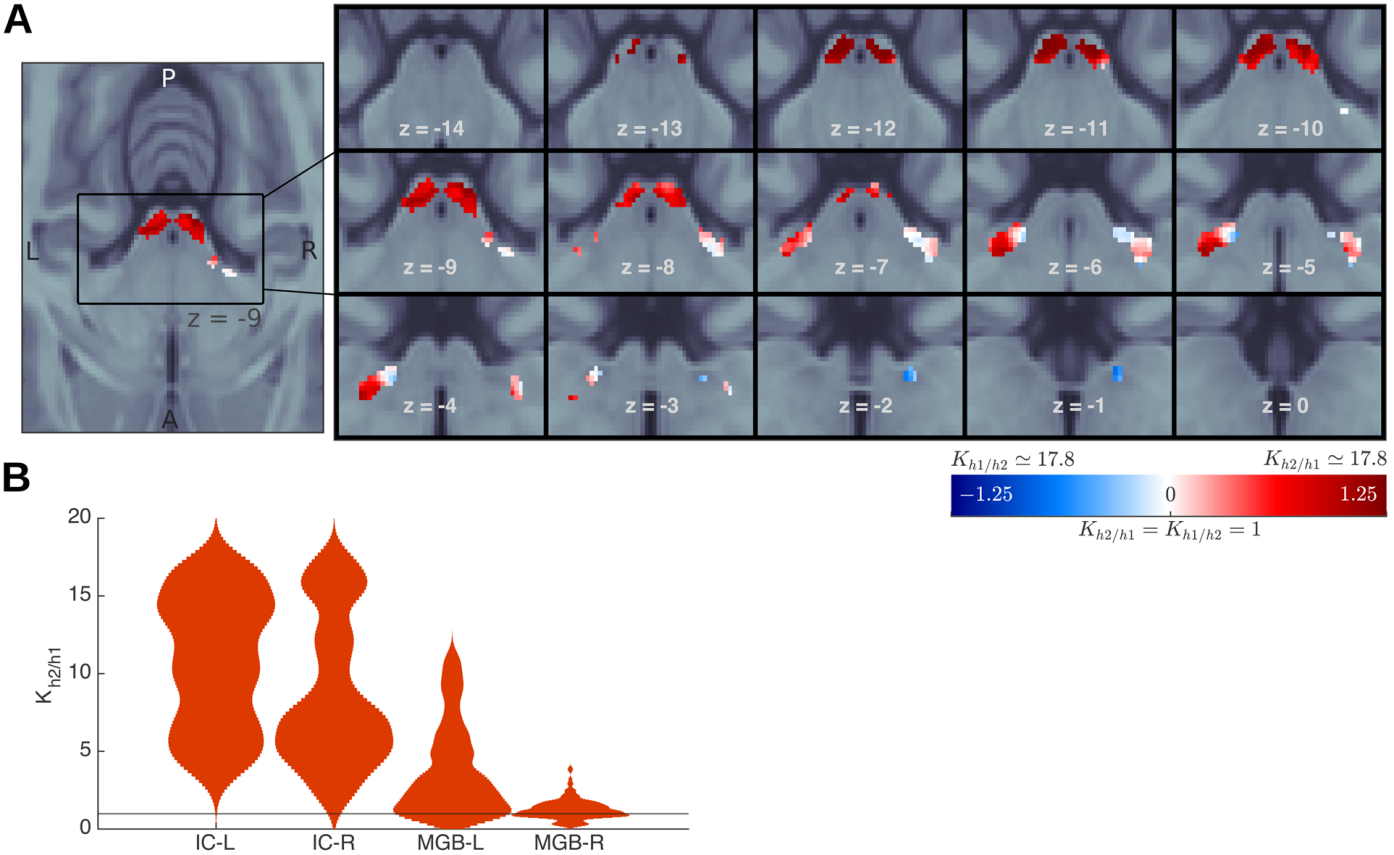
Bayesian model comparison. (A) Bayes’ factorKbetween h2 (predictive coding) and h1 (habituation) in each of the voxels of the subcortical ROIs in a logarithmic scale. Voxels with negativelog  Kvalues (K < 1; blue) are best explained by h1; voxels with positivelog  Kvalues (K > 1; red) are best explained by h2. Single-subjectKfactors are plotted inSupplementary Figure S5. (B) Kernel-density estimations of the distribution ofKfor the model comparison h2/h1 across voxels (i.e., one sample per voxel). SeeSupplementary Figure S3for a replication of these results, obtained using the mathematical definitions of the models ofTabas et al. (2020).

H2 was the best explanation for the data in the majority of voxels of the four ROIs (Fig. 7): h2 was more likely than h1 in all voxels of the left and right IC, and in 85% and 61% of the voxels of the left and right MGB, respectively (see also results from an alternative BMC analysis based onTabas et al. (2020)inSupplementary Fig. S3).

To test whether the effect was present at the single-subject level, we computedKindependently for each subject in the subject-specific bilateral IC and MGB. Since we performed the group analyses over the entire ROIs (and not only the SSA regions), here we used the full anatomical ROIs of each participant. We measured for how many voxels within each participant h2 was the better explanation of the data (see full results inSupplementary Fig. S5). In 15 of the 18 participants (all but subjects 3, 5, and 18), there were more voxels for which$K>\sqrt{10}$than for which$K<1/\sqrt{10}$; namely, more voxels for which there was substantial evidence in favour of h2 than for h1. These single-subject level results confirmed that responses do not simply habituate to successive repetitions of the tone but that, as hypothesised by predictive coding, they are also strongly affected by the subjective expectations of the listeners.

### 3.6 FM-sweeps are encoded as prediction error in primary and secondary MGB

The auditory pathway is anatomically subdivided into two sections: the primary (lemniscal) or secondary (non-lemniscal) pathways. The primary pathway is characterised by neurons that carry auditory information with high fidelity and it is generally regarded as responsible for the transmission of bottom-up sensory input (Hu, 2003). The secondary pathway has wider tuning curves and it is generally regarded as responsible for the integration of contextual and multisensory information (Hu, 2003).

Both IC and MGB comprise regions that participate in both, the primary and secondary pathways (Hu, 2003). The primary subdivision of the IC is its central nucleus, while the cortices constitute the secondary subdivisions. The primary subdivision of the MGB is its ventral section, while the medial and dorsal sections constitute the secondary subdivisions.

In rodents, SSA and prediction error to pure tones are significantly stronger in secondary subdivisions (e.g.,Parras et al., 2017). In humans, prediction error is similarly strong in primary and secondary MGB for pure tones (Tabas et al., 2020). Here, we test for differential representations of prediction error to FM-sweeps in MGB.

Distinguishing between the primary and secondary subsection of the IC and MGB non-invasively is technically challenging (Moerel et al., 2015). A recent study (Mihai et al., 2019) distinguished two distinct tonotopic gradients of the MGB. The ventral tonotopic gradient was identified as the ventral or primary (vMGB) subsection of the MGB (seeFig. 8A, green). Although the parcellation is based only on the topography of the tonotopic axes and their anatomical location, the region is the best approximation to-date of the vMGB in humans. No parcellation of the IC is available to-date.

**Fig. 8.**
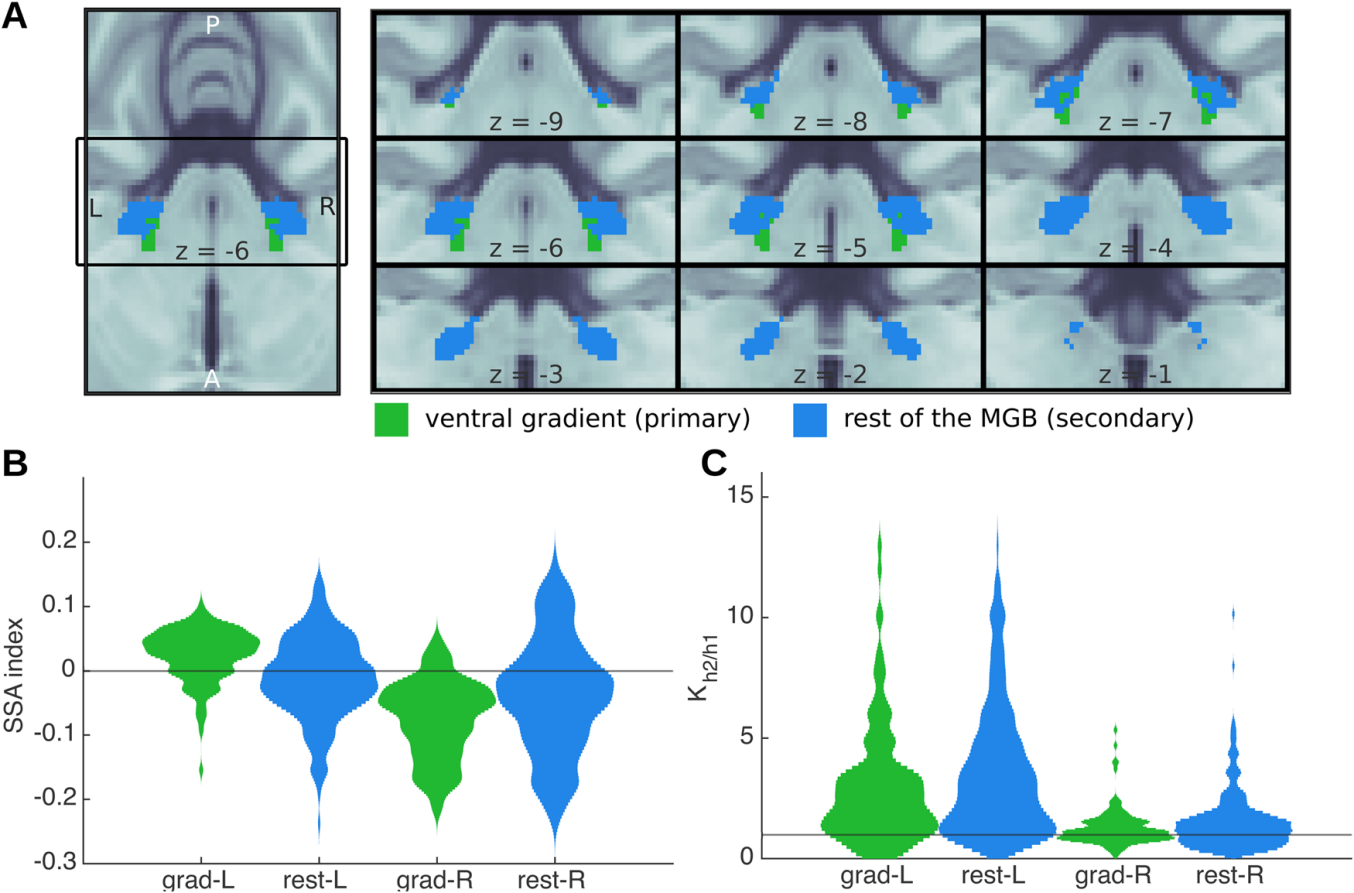
Analyses of BOLD responses in ventral MGB. (A) Masks fromMihai et al. (2019)of the ventral MGBs (green); blue indicates the remainder of the anatomical MGB ROIs. (B) The distribution of the SSA indexSIacross each of the two subdivisions of the MGB ROIs;SI>0is usually interpreted as SSA in the animal literature (Ulanovsky et al., 2003). (C) Histograms showing Bayes’ factor K for the comparison between h2 and h1 (Fig. 1F) in each of the subdivisions. No systematic functional differences are apparent between primary and secondary MGB.

Both primary and secondary subdivisions of bilateral MGB showed SSA. SSA strength was measured in each voxel using the SSA index (Eq. (1)). Distributions of theSIacross the voxels of each of the subdivisions were comparable in both hemispheres (Fig. 8B), demonstrating that SSA is not confined to nor stronger in the secondary MGB.

Predictive coding (h2) was the best explanation for the responses in 84% and 87% in the two subdivisions of the left MGB, and in 58% and 64% of the primary and secondary subdivisions of the right MGB, demonstrating the encoding of prediction error to FM-sweeps in both, primary and secondary subdivisions of bilateral MGB. Moreover, the distributions of the Bayes’ factorKbetween the predictive coding (h2) and adaptation (h1) hypotheses were comparable across subdivisions (Fig. 8C).

### 3.7 Prediction error to FM-sweeps and pure tones has similar topographic distributions in the IC

To study whether the same neural populations are in charge of encoding prediction error to FM-sweeps and pure tones, we compared the topographic distribution of the Bayes’ factorKbetween the h2 and h1 in our data with the topographic distribution of the Bayes’ factorKwe obtained in a previous experiment, where we measured BOLD responses to the same experimental paradigm as here but using pure tones (Tabas et al., 2020). We computed the correlation between bothKacross voxels of each of the four ROIs, as defined by the anatomical atlas fromSitek et al. (2019). To ensure that the analyses were comparable across studies, we ran a second Bayesian Model Comparison analysis on the current data using the same model definitions as inTabas et al. (2020)(seeSupplementary Fig. S3).

Distribution ofKto both families of stimuli was strongly correlated across voxels of bilateral IC (left,ρ=0.47,$p=4\times 10^{-9}$; right,ρ=0.34,$p=3\times 10^{-5}$;p-values corrected for 4 comparisons), but not across voxels of the MGBs (left,ρ=−0.11,p=0.22; right,ρ=0.15,p=0.1; uncorrectedp-values). These results indicate that the topographic distributions of prediction error responses are similar across stimulus modalities.

## 4 Discussion

The effects of expectations on sensory processing are readily evident in our daily lives. However, the neural mechanisms underlying the integration of expectations at early stages of the acoustic processing pipeline are poorly understood. Here, we have investigated how fast FM-sweeps, an important dynamic component of natural sounds, are encoded in the human subcortical auditory pathway, and how the subjective expectations of the listener influence their processing. Our study provided four main findings: first, we showed that the human IC and MGB comprise FM-direction and FM-rate selective neuronal populations. Second, we showed that responses in IC and MGB were driven by subjective expectations of the participants, demonstrating that the IC and MGB are integrated in a global network of predictive coding. The findings were robust and present at the single-subject level, demonstrating the generalisation power of the result. Third, we showed that the expectations determined the responses to FM-sweeps in primary and secondary subdivisions of bilateral MGB. Last, we showed that the topographic distribution of neural populations encoding the FM-sweeps as prediction error was similar to that of pure tones in the IC.

Combined, our results provide first demonstration that the human IC and MGB are actively engaged in the predictive processing of dynamic stimuli that transcend the tonotopic static representation: fast FM-sweeps. This confirms the long-standing hypothesis that predictive coding combines high-level expectations with the exquisite temporal properties of the subcortical auditory pathway to promote the encoding of dynamic low-level features (von Kriegstein et al., 2008;Yildiz et al., 2013). This mechanism might be responsible for boosting encoding efficiency and aiding, for example, speech recognition.

Neurons that respond selectively to FM-direction and FM-rate have been located in rodents in the IC (Geis & Borst, 2013;Hage & Ehret, 2003;Li et al., 2010), MGB (Kuo & Wu, 2012;Lui & Mendelson, 2003), and auditory cortex (Issa et al., 2016;Trujillo et al., 2013;Ye et al., 2010;Zhang et al., 2003). In contrast, FM-selectivity has been reported in humans in auditory cortex (Okamoto & Kakigi, 2015) or higher-order areas of the cerebral cortex (Hsieh et al., 2012;Joanisse & DeSouza, 2014). One previous study (Chandrasekaran et al., 2012) showed that auditory training improves encoding of rising FM-sweeps in the IC as measured by the frequency-following-response (Coffey et al., 2019), supporting the active involvement of the IC in the processing of FM. Here, we have established that neural populations in the human IC and MGB show SSA to FM-direction and FM-rate; since our FM-sweeps were matched in duration, pitch, and expected elicited activity along the tonotopic axis, our results extend previous findings providing first evidence for FM-direction and FM-rate selectivity in the human subcortical auditory pathway.

Animal studies have extensively shown that the SSA index to pure tones in IC and MGB increases with increasing rarity and frequency difference of the deviant with respect to the standard (Anderson et al., 2009;Antunes & Malmierca, 2011;Antunes et al., 2010;Ayala et al., 2013,^2015^;Duque & Malmierca, 2015;Duque et al., 2016;Malmierca et al., 2009;Zhao et al., 2011). These studies implicitly assume that sensory neurons form expectations based on the local statistics of the stimuli. This form of predictive coding, which we call*local*(Tabas & von Kriegstein, 2021a), is difficult to disambiguate from passive effects of neural habituation: Modelling studies have demonstrated that identical phenomenology can be produced by synaptic fatigue without the need of maintaining additional internal generative models (Eytan et al., 2003;Mill et al., 2011,^2012^). Manipulating expectations orthogonally to stimulus regularities is the only way to assess if prediction error is computed with respect to a global model of the sensory world (Tabas & von Kriegstein, 2021a).

To date, the only evidence (seeTabas & von Kriegstein, 2021afor a review) that subcortical nuclei encode stimuli according to subjective expectations independently of stimulus regularities was provided by our previous study on pure tones in human IC and MGB (Tabas et al., 2020). Here, we used fast FM-sweeps that were explicitly designed to elicit the same activation across the tonotopic axis (Tabas & von Kriegstein, 2021b) to ensure that participants had to make use of FM-direction and FM-rate selective neurons to differentiate the deviant from the standards. The current findings demonstrate that the same principles apply to the encoding of dynamic FM-sweeps.

Our results also showed that the topographic distribution of voxels encoding pure tones and FM-sweeps according to the principles of predictive coding was highly correlated in the IC, but not in the MGB. This divergence might indicate a different functional role of the IC and the MGB with respect to both families of stimuli; however, it might also be caused by a greater variability in the anatomical location and orientation of the MGB across subjects (Moerel et al., 2015) and should be considered with caution.

The expectations induced by our paradigm are still far from the complexity of the predictive system putatively in charge of the processing of natural complex signals like speech. However, we speculate that an integrated inverted hierarchy could propagate linguistic predictions to the representational level of formant transitions (Friston et al., 2021;Tabas & von Kriegstein, 2021a;von Kriegstein et al., 2008), and use these predictions to compute prediction error in the IC and MGB.

The expectations induced by our paradigm are most likely generated in the cerebral cortex. However, since we optimised our paradigm to study prediction error rather than the generation of expectations, we cannot test whether the subcortical responses we measured are driven or not by corticofugal projections. This possibility would be consistent with the massive corticofugal connections from cerebral cortex to MGB and IC (Winer, 1984,2005b), and with results from animal studies where the deactivation of unilateral auditory cortex (Bauerle et al., 2011) or the thalamic reticular nucleus (Yu et al., 2009) led to reduction of SSA in the ventral MGB (but also see contradictory findings in non-lemniscal MGB (Antunes & Malmierca, 2011) and non-lemniscal IC (Anderson & Malmierca, 2013)).

The present and previous (Tabas et al., 2020) results demonstrate that the IC and the MGB encode auditory stimuli according to subjective expectations when the sensory signal is relevant for the listener’s task. This encoding strategy might 1) be general to sensory processing or specific to the processing of task-relevant stimuli; and 2) be particular of processing under abstract expectations that are explicitly known by the listeners or general to any expectation that could be inferred from exposure to the sensory input. Previous studies showed that the IC and the MGB adapt to stimulus regularities even in the absence of a task (e.g.,Cacciaglia et al., 2015). Whether abstract regularities also affect the encoding of task-irrelevant stimuli in the subcortical pathway is still an open question.

Despite the fact that the MGB is at a higher processing stage than the IC, we found similar prevalence of the predictive coding Bayesian model (h2) in both nuclei for FM-sweeps (Fig. 7) as well as for pure tones (Tabas et al., 2020). These results contrast with a study in rodents, concluding that the MGB encodes prediction error more strongly than the IC (Parras et al., 2017). We speculate that this fundamental difference is caused by the introduction of abstract rules in our paradigm. Since prediction error depends only on the local representation and the predictions (Friston, 2003), there is no reason for prediction error to vary across hierarchical stages that receive the same set of predictions and have comparable representation of the stimuli, as it is the case for FM-sweeps in IC and MGB (Kuo & Wu, 2012) (and also for pure tones (Hu, 2003)). Rodent studies use passive listening tasks where expectations are induced by repetition. Without an explicit high-level model, prediction error can only be computed with respect to local models that may vary in complexity across processing stages. A task involving stimuli that are represented differently in IC and MGB should shed light on the hierarchical role played by each of the two stages.

Previous studies on subcortical SSA rested almost exclusively on pure tones (Carbajal & Malmierca, 2018;Malmierca et al., 2015;Tabas & von Kriegstein, 2021a). Only three studies considered whether SSA generalised to other acoustic properties.Thomas et al. (2012)reported SSA to FM-rate in the IC of the big brown bat; however, since the authors used stimuli in the rate range of echolocation signals, it was unclear whether this behaviour would generalise to auditory FM.Gao et al. (2014)measured SSA using ramped and damped broadband noises in the IC, demonstrating that neurons in the IC adapt to intensity modulation. Last,Duque et al. (2016)measured SSA to intensity, and showed that neurons in the IC do not adapt to nominal loudness. Our findings complement these results showing that the human IC and MGB adapt to fast FM without loudness or spectral changes, and provides first evidence for SSA to acoustic properties other than pitch and loudness in the subcortical pathways.

We have argued that the response pattern in MGB and IC (Fig. 6) can be interpreted as the encoding of prediction error with respect to the subjective expectations of the participants. There are two conceivable alternative interpretations for the results: that responses are driven by attention-driven gain modulation, and that responses are driven by general habituation to auditory stimuli. The former view interprets the higher responses todev4anddev5as the result of a stronger attention of the participant to these positions, which are relevant to the task, and the lower responses todev6, which is fully expected, as the results of a reduction of attention. Previous fMRI studies have indeed shown that attended stimuli elicited higher BOLD responses in auditory cortex (Lee et al., 2014;Paltoglou et al., 2011), and to a much weaker extent also in the IC (Riecke et al., 2018;Rinne et al., 2007,^2008^;Varghese et al., 2015). However, this interpretation of our results is unsatisfactory because, first, we observe statistically significant differences in the BOLD responses todev4anddev5, although they are both equally relevant for the task. Second, we observed no systematic differences between responses todev6andstd2, whereas in a previous fMRI study deviants always elicited statistically significantly higher responses than standards (Cacciaglia et al., 2015). This was the case although the study had lower statistical power in comparison to our study and used passive stimulation. Only by interpreting the BOLD responses inFigure 6as prediction error with respect to the participant’s expectations we can explain the similar responses todev6andstd2in our paradigm.

The other conceivable interpretation is that the response pattern found in MGB and IC (Fig. 6) is driven by a kind of habituation that partially generalises to tones of other frequencies. This kind of*general*habituation has been reported in the human auditory cortex (e.g.,Rosburg et al., 2002). Sincedev6is preceded by five standards, it is plausible that general habituation incurs into lower responses todev6than todev5ordev4, which are preceded by four and three standards, respectively. However, this interpretation of our results is also unsatisfactory. First, the effect size of the reduction of the responses todev5with respect todev4isd≃1, and the effect of the response reduction fromdev5todev6is even stronger. If one more repetition of a standard was responsible for such a large reduction of the responses, we would expect the responses todev4, which is preceded by three standards, to be much smaller than the responses to the first standard. However, we observe similar responses todev4andstd0. Second, if there were strong general habituation effects capable of inducing a significant decrease in the BOLD responses in tones preceded by more than three standards, we would expect the responses to the standard preceding the deviant (std1) to elicit stronger responses than the standards following the deviant (std2). However, the results inFigure 6show no systematic differences in the responses tostd1andstd2.

It has been previously suggested that prediction error may be encoded exclusively in the non-lemniscal or secondary subdivisions of the IC and MGB (Ayala et al., 2015;Malmierca et al., 2015;Parras et al., 2017). In agreement with this hypothesis, SSA is stronger in secondary subdivisions of the rodent’s IC (Ayala & Malmierca, 2018;Ayala et al., 2015;Duque et al., 2014;Gao et al., 2014;Pérez-González et al., 2012) and MGB (Antunes & Malmierca, 2011;Antunes et al., 2010;Duque et al., 2014).

In contrast, our results indicated an apparent lack of specialisation across subdivisions of the MGB during the encoding of FM-sweeps: both subdivisions were similarly responsive to FM, and they both encoded FM as prediction error. Similar results were apparent in our previous study when we investigated the encoding of pure tones (Tabas et al., 2020). This lack of specialisation would fit with the idea that expectations are used in the subcortical pathways to aid encoding: to optimise the resources of the subcortical stations requires to make use of the narrow receptive fields of the primary subdivisions (Hu, 2003).

The fundamental difference between our results and the findings in animals might stem from a number of reasons. First, our design involved an active task: lemniscal pathways might only be strongly modulated by predictions when they carry behaviourally relevant sensory information. Second, the modulation of the subcortical auditory pathway might be fundamentally different in humans compared to other mammals, as they have to accomplish processing of such complex and dynamic signals as speech. Last, given the strength of the SSA effects reported in this study, it is possible that regions with weak SSA might have been contaminated with signal stemming from areas with strong SSA due to smoothing and interpolation necessary for the analysis of fMRI data.

Given the paramount role of predictions on sensory processing (Blank et al., 2018;Davis et al., 2011;Davis & Johnsrude, 2007;de Lange et al., 2018;Sohoglu et al., 2012), atypical predictive coding in the subcortical sensory pathway could have profound repercussion at the cognitive level (Diaz et al., 2012;McFadyen et al., 2020;Tabas & von Kriegstein, 2021a). For instance, developmental dyslexia has been attributed to altered adaption dynamics to stimulus regularities (Ahissar et al., 2006;Chandrasekaran et al., 2009;Perrachione et al., 2016), altered responses in the left MGB (Chandrasekaran et al., 2009;Diaz et al., 2012), and atypical left hemispheric cortico-thalamic pathways (Müller-Axt et al., 2017;Tschentscher et al., 2019). Understanding the mechanisms underlying the predictive processing of dynamic acoustic features in subcortical sensory pathways is an essential prerequisite to understand dysfunction.

## Supplementary Material

Supplementary Material

## Data Availability

Derivatives (beta maps and log-likelihood maps, computed with SPM) and all code used for data processing and analysis are publicly available inhttps://osf.io/f5tsy/.
