# Supplementary material for: Fast frequency modulation is encoded according to the listener expectations in the human subcortical auditory pathway
Source: Imaging Neurosci (Camb). 2024 Sep 19;2:imag-2-00292. doi: 10.1162/imag_a_00292 (PMC12290582; doi:10.1162/imag_a_00292)
Supplement: Supplementary Material [file imag_a_00292-supp.pdf]

Supplementary Materials for "Fast frequency modulation is encoded according to the listener expectations in the human subcortical auditory pathway"

Alejandro Tabas[1,2,3], Stefan Kiebel[3], Michael Marxen[4,5], and Katharina von Kriegstein[3,2]

[1]Basque Center on Cognition, Brain, and Language, San Sebastian, Spain
[2]Max Planck Institute for Human Cognitive and Brain Sciences, Leipzig, Germany
[3]Department of Psychology, Technische Universität Dresden, Dresden, Germany
[4]Department of Psychiatry, Technische Universität Dresden, Dresden, Germany
[5]Neuroimaging Center, Technische Universität Dresden, Dresden, Germany

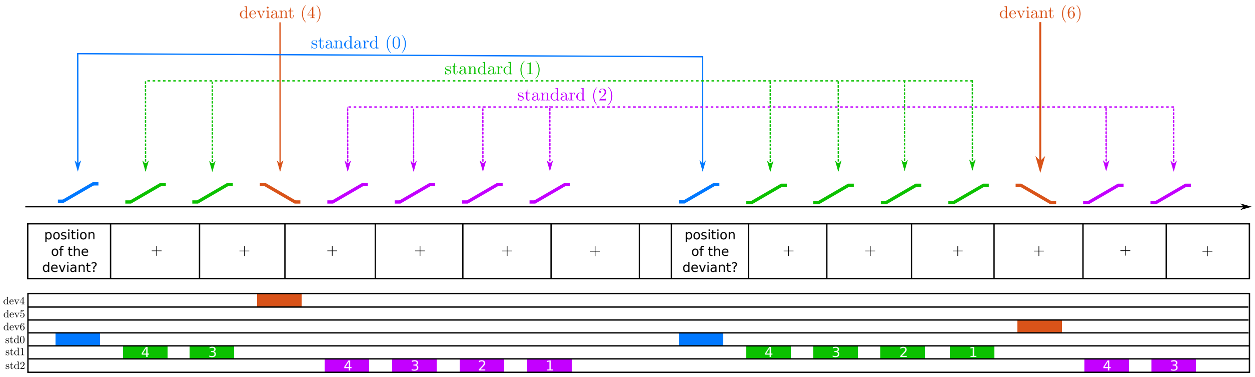

Figure S1: **Schematic of the GLM's design matrix.** An example of the GLM design matrix section corresponding to the regressors of interest prior to the convolution with the haemodynamic response function. The example includes two trials with two different deviant positions. The six regressors of interest were standard 0, standard 1, standard 2, deviant 4, deviant 5 (not shown), deviant 6. The standards were parametrically modulated and the modulation was equal to the inverted index of the standard within the sequence (i.e., 4 for the first *std*1, 3 for the second *std*1, etc; note that, since the modulation was mean-corrected before the fitting of the GLM, the absolute values of the modulation are not relevant

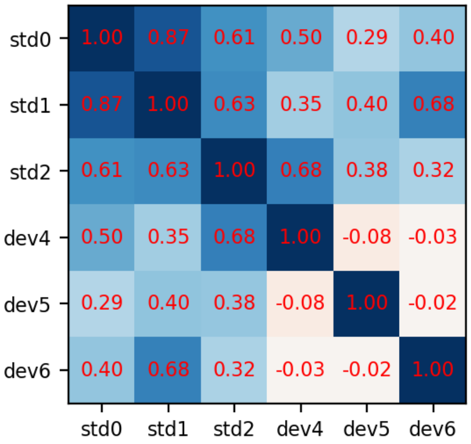

Figure S2: **Correlations between the regressors fitted in the GLM for an example trial.** Each point in the matrix corresponds to the Pearson's correlation of the time series of two regressors (described in the x- and y- axes) along the entire run, convoluted with the cannonical haemodynamic response function [Glover, 1999].

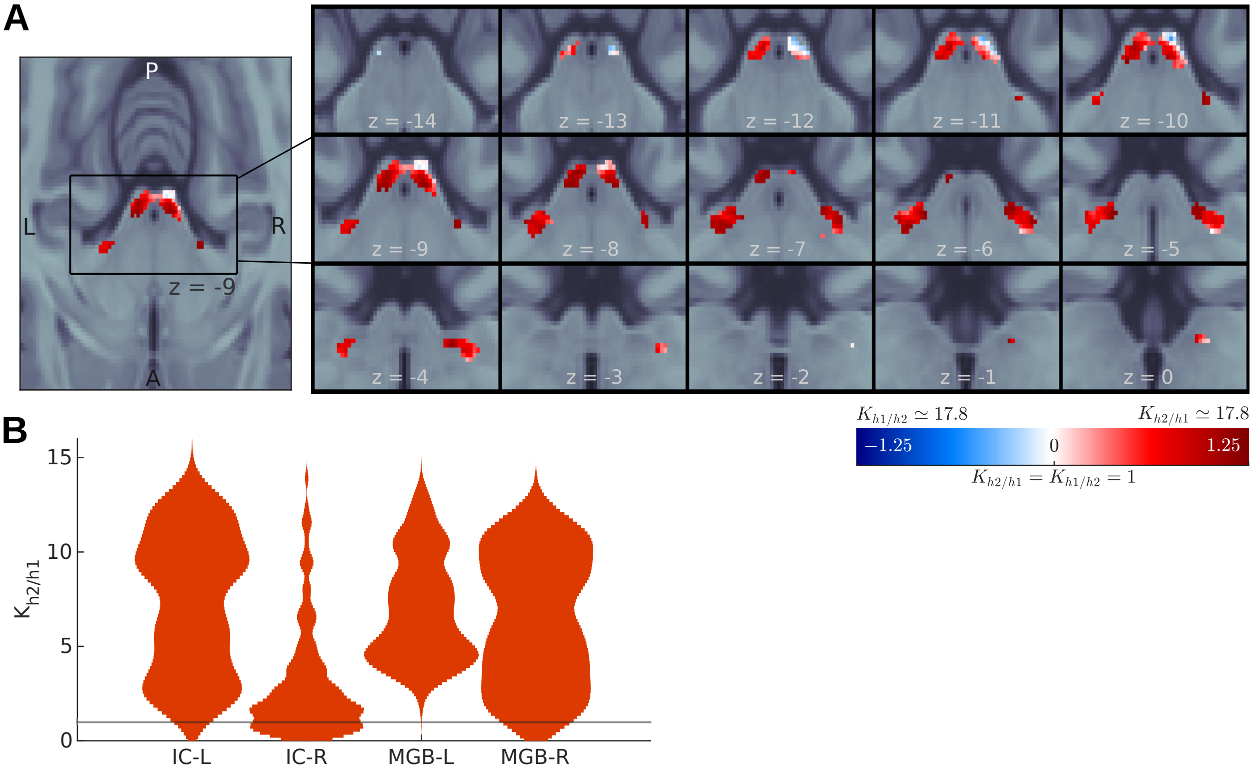

Figure S3: **Bayesian model comparison analysis with using alternative models.** This figure replicates Figure 7 but using the model definitions of [Tabas et al., 2020] instead of the definitions of Table 1. Both sets of results confirm the encoding of prediction error to FM-sweeps in bilateral IC and MGB. As expected, the summary statistics and proportion of voxels significantly explained better by h2 vary across the two analyses. This is a natural reflection that K is a random variable: its nominal values are expected to vary across model definitions and noise distributions of the data. Note that K is not to be interpreted as a measure of the strength of the effect, but as a measure on the confidence of the higher explanatory power of a model over the other. A) Bayes' factor K between h2 (predictive coding) and h1 (habituation) in each of the voxels of the subcortical ROIs in a logarithmic scale. Voxels with negative $\log K$ values ($K < 1$; blue) are best explained by h1; voxels with positive $\log K$ values ($K > 1$; red) are best explained by h2. B) Kernel-density estimations of the distribution of K for the model comparison h2/h1 across voxels (i.e., one sample per voxel).

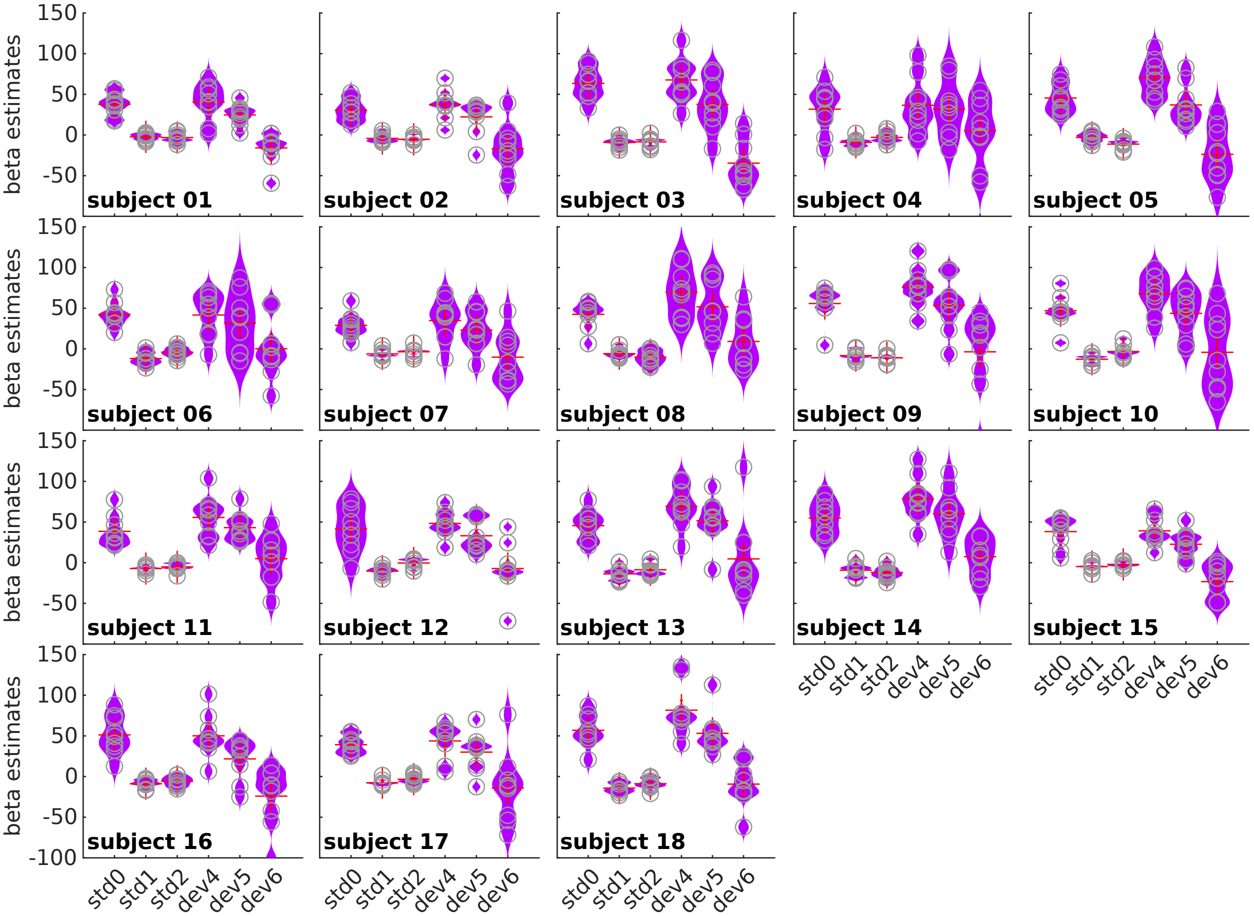

Figure S4: **Summary BOLD responses at the single-subject level.** Average z-score in each of the four subcortical ROIs to the different regressors. Violin plots are kernel density estimations of the distribution of z-scores, averaged over voxels and runs of each ROI. Each distribution holds 9 samples, one per run. Black error bars show the mean and standard error of the distributions. Unlike Figure 6, the analysis included the full subcortical ROIs (as computed using the functional localiser of each participant) rather than the subset of voxels that showed SSA (i.e., the SSA ROIs).

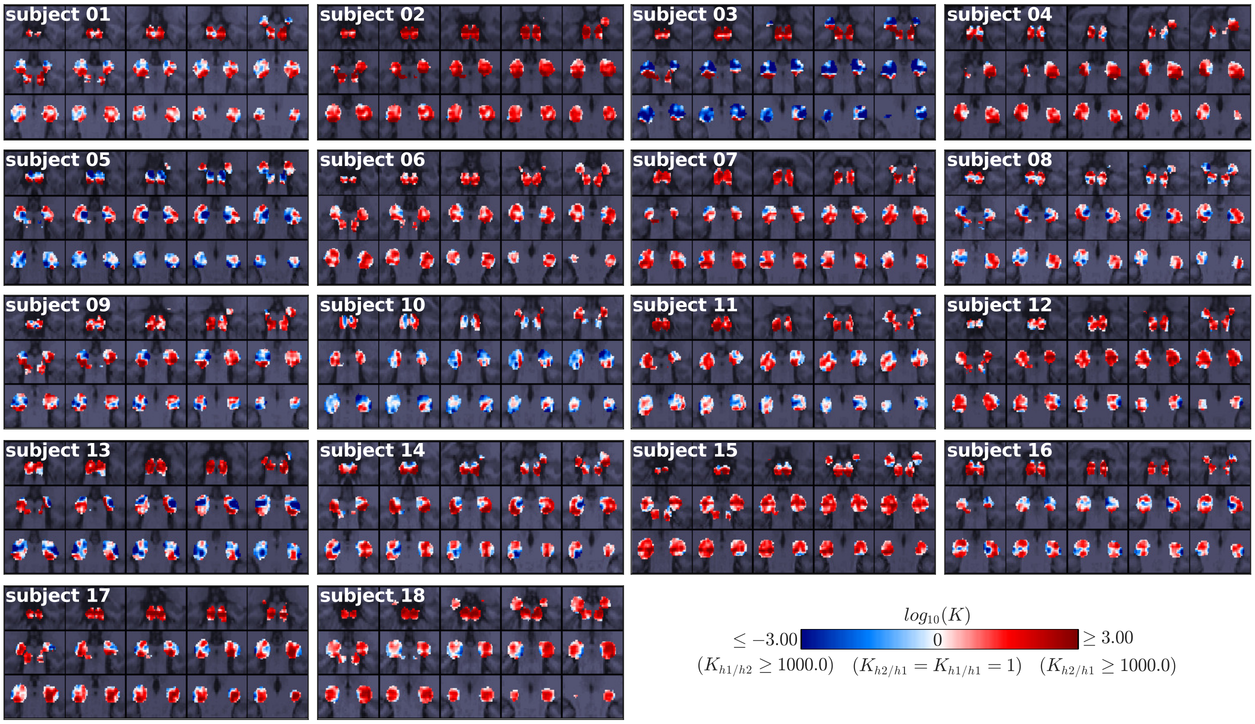

Figure S5: **Bayesian model comparison at the single-subject level.** A) Bayes' factor K between h2 (predictive coding) and h1 (habituation) in each of the voxels of the subcortical ROIs for each subject in a logarithmic scale. Voxels with negative $\log K$ values ($K < 1$; blue) are best explained by h1; voxels with positive $\log K$ values ($K > 1$; red) are best explained by h2. Unlike Figure 7, the analysis included the full subcortical ROIs (as computed using the functional localiser of each participant) rather than the subset of voxels that showed SSA (i.e., the SSA ROIs).
